# Proteomic Study of Differential Protein Expression in Mouse Lung Tissues after Aerosolized Ricin Poisoning

**DOI:** 10.3390/ijms15057281

**Published:** 2014-04-28

**Authors:** Zhendong Guo, Chao Han, Jiajun Du, Siyan Zhao, Yingying Fu, Guanyu Zheng, Yucheng Sun, Yi Zhang, Wensen Liu, Jiayu Wan, Jun Qian, Linna Liu

**Affiliations:** 1 Institute of Military Veterinary, Academy of Military Medical Sciences, 666 West Liuying Road, Changchun 130122, Jilin, China; E-Mails: guozd@foxmail.com (Z.G.); zhaosy@foxmail.com (S.Z.); ycsun_73@126.com (Y.S.); yizhang818@163.com (Y.Z.); wsliu_1101@126.com (W.L.); jywan_1973@163.com (J.W.); 2 263 Clinical Department of General Hospital of Beijing Military Region, Beijing 101149, China; E-Mail: hc1345@126.com; 3 Medical Information Center of Chinese PLA General Hospital, Beijing 100853, China; E-Mail: spna7@126.com; 4 College of Animal Science and Technology, Jilin Agricultural University, Changchun 130122, Jilin, China; E-Mails: fyyinging@163.com (Y.F.); zhengguanyu1@163.com (G.Z.)

**Keywords:** ricin, aerosol, lung injury, proteomics

## Abstract

Ricin is one of the most poisonous natural toxins from plants and is classified as a Class B biological threat pathogen by the Centers for Disease Control and Prevention (CDC) of U.S.A. Ricin exposure can occur through oral or aerosol routes. Ricin poisoning has a rapid onset and a short incubation period. There is no effective treatment for ricin poisoning. In this study, an aerosolized ricin-exposed mouse model was developed and the pathology was investigated. The protein expression profile in the ricin-poisoned mouse lung tissue was analyzed using proteomic techniques to determine the proteins that were closely related to the toxicity of ricin. 2D gel electrophoresis, mass spectrometry and subsequent biological functional analysis revealed that six proteins including Apoa1 apolipoprotein, Ywhaz 14-3-3 protein, Prdx6 Uncharacterized Protein, Selenium-binding protein 1, HMGB1, and DPYL-2, were highly related to ricin poisoning.

## 1. Introduction

Ricin, a glycoprotein derived from the castor bean, is extremely toxic if taken in through oral, respiratory, or percutaneous routes. In fact, ricin has been used in multiple assassination attempts because of its high toxicity [1]. Ricin is a putative lethal agent of biological warfare because it can be easily extracted from the waste mash of castor oil production and from the widely available castor beans. With a molecular weight of 62-kDa, ricin consists of two glycoprotein chains linked through a disulfide bridge: 32-kDa A chain and 34-kDa B chain. The 34-kDa B chain, a lectin that binds primarily to galactose-containing surface proteins, facilitates the internalization of the 32-kDa A chain, the toxic moiety. Following endocytosis and retrograde transfer through the endosome, Golgi apparatus and endoplasmic reticulum, the A chain of ricin enters the cytosol where it depurinates a single adenine (A4324 in mouse) in the 28S rRNA ribosomes. The depurination of the A4324 is directly responsible both for the inhibition of protein translation and for the initiation of upstream events that lead to inflammatory responses [2–5].

Rauber *et al.* documented more than 750 cases of accidental or deliberate ricin intoxication from castor bean ingestion, with 14 resulting in fatality [6]. The ingestion fatal dose is 5–6 castor beans for children and 20 beans for adults [7]. Although considerably less potent than botulinum neurotoxins and staphylococcal enterotoxins, ricin is still considered a significantly potential biological weapon because of its stability and worldwide availability as a by-product of castor oil production. In addition, it has been associated with several terrorist actions and therefore is a potential bioterrorism agent [7]. The Centers for Disease Control and Prevention (CDC) of U.S.A categorizes ricin as a Category B Agent (the second-highest priority), as it can be moderately easily disseminated, leading to low mortality but moderate to high morbidity. It can be injected into a subject, be used to contaminate food and water, or be dispersed as an aerosol. Ricin poisoning can cause severe tissue damages and inflammatory reactions and can result in death [8]. The initial symptoms of ricin poisoning include nausea, vomiting, diarrhea, and abdominal pain. In severe poisoning, the symptoms can progress to gastrointestinal bleeding with necrosis of the liver, spleen and kidney, and even cardiovascular collapse [9]. If inhaled, ricin can cause death within 36–48 h because of the failure of the respiratory and circulatory systems [10]. As a toxin, ricin is not a disease-causing agent. Therefore, it is not contagious and cannot be spread from person to person through casual contacts [11].

While the damage on humans through aerosol exposure was not well reported, lesions induced by oral and parenteral exposures are consistent with those from animal studies, suggesting that the same would hold true for aerosol exposures. Studies on mice demonstrated that aerosolized ricin is deposited in trachea and lungs. Pulmonary deposition highly depends on aerosol particle size, which profoundly affects the mortality rate in the animal model [12,13]. Aerosolized ricin exposure leads to weakness, fever, cough, and pulmonary edema symptoms within 18–24 h and severe respiratory distress and even death within 36–72 h [14]. There is no effective treatment for ricin poisoning, and no vaccine or prophylactic antitoxin available for human either [15]. Studies in rodents and nonhuman primates have demonstrated that ricin delivered into the pulmonary system leads to acute lung injuries and symptoms which resemble acute respiratory distress syndrome (ARDS) [16]. When administered into the animal lungs, ricin induces a rapid and massive migration of inflammatory cells [17]. Another early consequence of ricin exposure is the activation of a rapid-acting primary transcription factor that induces expressions of genes encoding several pro-inflammatory cytokines and chemokines [5,18,19]. Primary human airway epithelial cells and primary murine macrophages respond to ricin *in vitro* through the activation of both MAPK and NF-κB [20], but the specific cell types responsible for ricin’s lethal inflammatory effects *in vivo* remain unclear.

In this study, proteomic technique was used to explore the mechanism of ricin aerosol exposure-induced lung injury in mice. So far, there are only limited studies reported on the toxicology of inhaled ricin. Since there is no effective drug or clinically approved vaccine for ricin poisoning, a better understanding of the mechanism of the ricin toxicity can offer new directions to develop new treatments for the respiratory poisoning caused by ricin aerosol.

## 2. Results and Discussion

### 2.1. Animal Models

**Figure 1.**
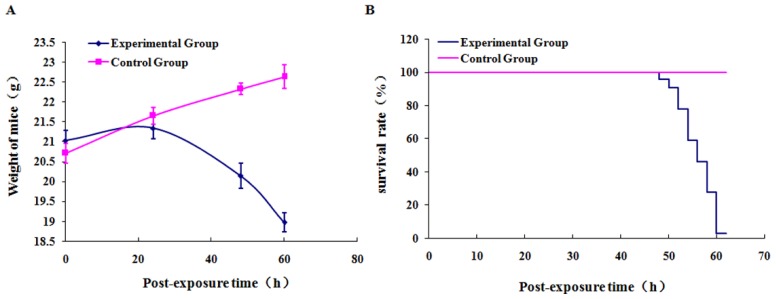
Changes in the weights (**A**) and survival rates (**B**) of mice after ricin aerosol exposure.

Ricin aerosol particles ranged from 0.7 to 1 μm (in diameters). The average weight of the mice in Group E (experimental group, ricin exposure) increased gradually during the first 24 h post-exposure, but the growth rate was lower than that of Group C (Figure 1). After 24 h, the average weight of the mice in Group E began to drop. 24–48 h after the exposure, more than half of the mice in Group E showed malaise, lower mobility, somnolence, and dark eyes. 48–60 h after the exposure, the mice in Group E stopped eating, exhibited even lower mobility, had messy hair on their back, and showed shortness of breath accompanied with murmurs. Their eye color deepened and tail veins turned purple. Then the mice started to die one after another, with the survival rate falling rapidly. 62 h after the exposure, 97% of the mice died from difficulty in breathing and transient mania (Figure 1). In comparison, no adverse reaction was observed for the mice in Group C (control group, saline exposure) during the whole process.

### 2.2. Pathological Observation

**Figure 2.**
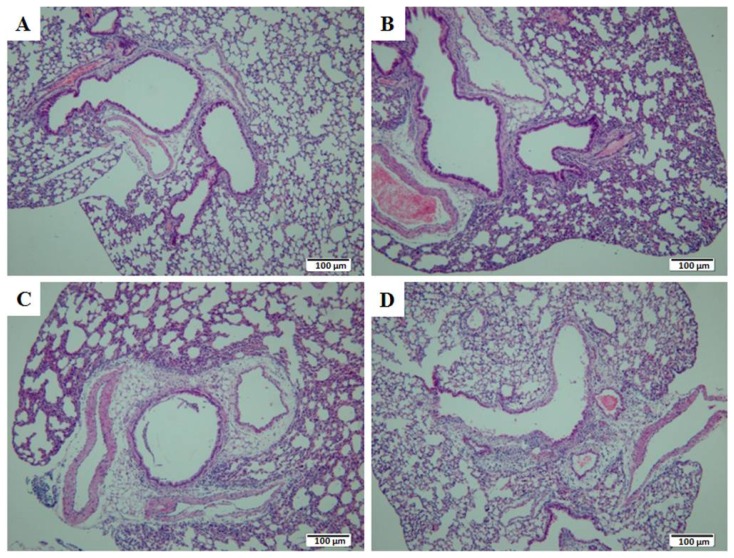
Pathological observation of the lung tissues of mice at 12 h (**B**), 24 h (**C**) and 48 h (**D**) post-exposure to ricin. The lung tissues of mice in Group C were used as control (**A**).

Pathological observations of lung and bronchiole tissues were shown in Figure 2. No obvious pathological change was observed in lung and bronchiole tissues of mice from both Group C (Figure 2A) and Group E at 12 h post-exposure to aerosolized ricin (Figure 2B). While at 24 h post-exposure, bronchiole mucous epithelium began to desquamate, and lymphocytes and macrophages were scattered (Figure 2C). At 48 h post-exposure, bronchiole mucous epithelium was damaged obviously and partially desquamated, and bronchiole wall was attached with an mixture of inflammatory exudate and necrotic epithelium, accompanied with submucosal edema, infiltration of lymphocytes and macrophages (Figure 2D).

### 2.3. 2-DE and Mass Spectrometry Analysis

**Figure 3.**
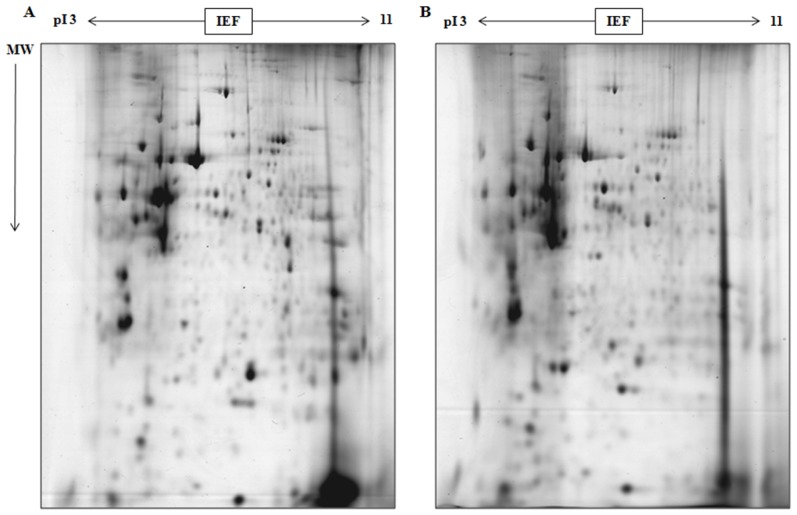
Proteomic analysis of lung tissues from control group and experimental group (48 h post-exposure) using 2-DE gels. Proteins on 2-DE gels of control group (**A**) and experimental group (**B**) were visualized using Coomassie blue staining.

**Figure 4.**
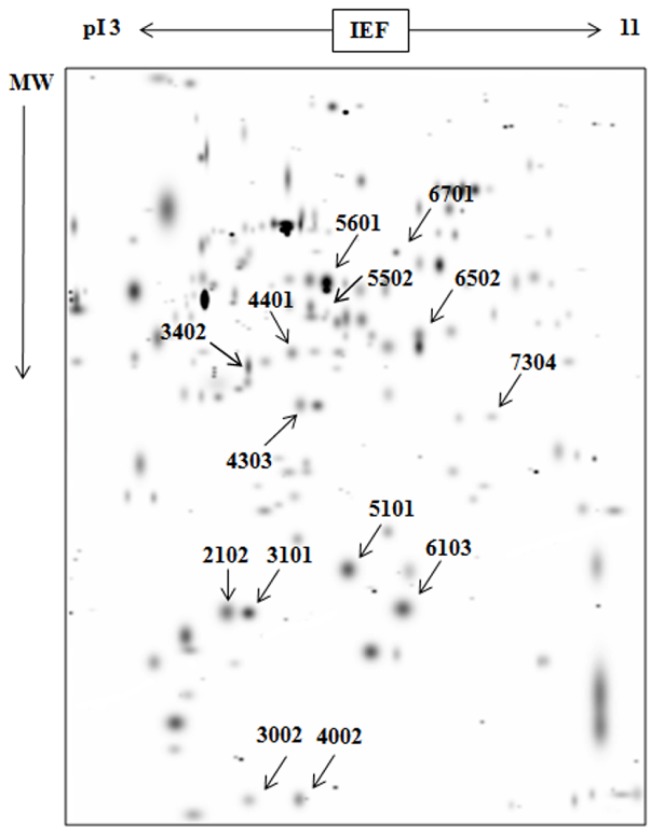
Differential expression of proteins analyzed using PDQuest. PDQuest software (BIO-RAD) was used to analyze the scanned 2-DE gel image. The spots representing significantly different protein expression were selected (Table 1) for further mass spectrometric analysis.

Following 2-DE (Figure 3) and software analysis (Figure 4, Table 1), 14 protein spots were selected and enzymatically hydrolyzed. MALDI-TOF/MS identification was performed on these 14 proteins, with 8 were successfully identified. SSP2102 and SSP3101 were Apoa1 apolipoprotein. SSP3402, SSP4401 and SSP6502 were Alb serum albumin, while SSP5103 and SSP6103 were Prdx6 uncharacterized protein (Table 2).

In this study, an animal model for ricin aerosol exposure was established and the differential protein expressions in lung tissues were determined using proteomic analysis. Eight proteins associated with respiratory injury were successfully identified, which were involved in functions such as cellular transportation, cytoskeletal development, signaling pathways and anti-oxidation. The causality relationship between ricin poisoning and the biological functions of proteins Apoa1 apolipoprotein, Ywhaz 14-3-3 protein, Prdx6 Uncharacterized Protein, Selenium-binding protein 1, HMGB1, and DPYL-2 was discussed.

Apolipoprotein A-I (Apoa1) is one of the apolipoproteins and the main component of high density lipoprotein (HDL). The main function of Apoa1 is to stabilize the structure of lipoproteins and to transport lipids. It also activates lipoprotein metabolic enzymes and recognizes the corresponding receptors. Apoa1 is synthesized primarily in the liver, and partly in the small intestine. The expression level of Apoa1 is closely related to the liver functions [21]. One typical symptom of ricin poisoning is the damage to organs such as liver and kidney. The lower Apoa1 expression after ricin aerosol exposure, suggests that Apoa1 might be associated with the systemic organ failure and liver tissue lesions following the acute lung injury resulted from ricin aerosol poisoning.

Ywhaz 14-3-3 protein belongs to a highly conserved acidic protein family and has a molecular weight of about 30 KDa. There are seven isotypes in eukaryotic cells, including β, γ, ɛ, ζ, η, σ and τ isotypes. 14-3-3 protein can be synthesized by all organs in but is abundant in the nervous system, distributed mainly in the cytoplasm. Currently over 100 proteins have been found to interact with 14-3-3 protein. Those 100 proteins are involved in cell division, signal transduction, apoptosis and membrane transport. 14-3-3 proteins can inhibit or enhance the activity of target proteins, protect target proteins against protease and phosphatase, and mediate the interactions of two target proteins as either adapter proteins or scaffold proteins [22]. Recently, one report pointed out that the expressions of 14-3-3 proteins were significantly elevated in the cerebrospinal fluid of patients with Alzheimer’s disease [23]. Chandra’s experiment showed that the expression of Ywhaz 14-3-3 protein is down-regulated when rats’ macrophages are poisoned with anthrax toxin [24]. Our results showed increased expression of 14-3-3 protein, which might be closely related to organ lesions and pathological damages following ricin poisoning. Typical toxic effects of ricin included cell apoptosis and parenchyma organ damage, combining with 14-3-3 protein’s biological functions, our studies thus provided the potential cues to further indentify that if there existed interactions between ricin and 14-3-3 protein.

Peroxiredoxins (Prdx) belong to a widely expressed non-selenium-dependent peroxidase superfamily only discovered over the last decade. Based on the number of cysteine residues, Prdx can be classified as Prdx1-5 (1 unit of cysteine residue) or Prdx6 (2 units). Prdx6 is a bifunctional protein with both phospholipase A2 activity and glutamate/cysteine aminopeptidase peroxidase activity [25]. Recent studies have confirmed that Prdx6 is highly expressed in Clara cells and alveolar type II epithelial cells in the lungs, and plays a protective role in the oxidative damage on lungs [26]. In this study, Prdx6 expression significantly decreased, suggesting the importance of this protein in the ricin-induced lung injury.

Selenium-binding protein (SBP) is a protein that binds to selenium in the body and is highly expressed in lung, liver, pancreas and thyroid. SBP1 can prevent peroxide-induced DNA damage, boost the expression of P53 and Cox genes, induce apoptosis, and inhibit tumor cell proliferation. However, little is known about the mechanism of the SBP1 bioactivity *in vivo*. Studies have shown that SBP1 expression is reduced in a variety of tumor cell lines, making SBP1 a diagnostic indicator for malignancy [27]. In this study, SBP1 expression decreased after lung injury, which may be related to DNA damage and apoptosis resulted from ricin poisoning. Further studies are needed to determine the mechanism of SBP1.

High mobility group protein B1 (HMGB1) is a non-histone chromatin-binding protein, and a highly phosphorylated protein. HMGB1 is involved in gene transcription and DNA replication, repair and recombination by assembling and stabilizing the nucleosomes and binding to transcription factors [28]. Recently, HMGB1 has been found to mediate the lethal systemic inflammatory response. Studies have shown that inflammatory cytokines TNF-α or IL-1 can stimulate monocyte-macrophages to release HMGB1, which reversely stimulates the secretion of inflammatory cytokines and induces the chemotaxis of neutrophils. HMGB1 also plays an important role in the development of anti-virus vaccines [29]. Ricin poisoning can induce systemic inflammatory response, leading to inflammatory cytokines “storm”. The expression level of HMGB1 significantly increased in the experimental group, which might be the direct result of this inflammatory response.

Dihydropyrimidinase-related protein 2 (DPYL-2), also known as CRMP-2, belongs to the dihydropyrimidinase-related protein family. This protein is involved in the neuronal differentiation process and plays a key role in T lymphocyte polarization and migration [30]. Lung is not only an important respiratory organ but also an endocrine organ. Cell populations with morphological and functional characteristics of both endocrine cells and nerve cells are found in most parts of lung from the trachea and bronchus to the alveoli. Ricin poisons animals through the aerosol route, inducing apoptosis and nerve damage. However, it remains unclear whether these symptoms are caused by the indirectly induced nerve damage from the solid organ injuries or by the direct damages from ricin on the nerve system.

Future studies will focus on screening and identifying these differentially expressed proteins that might be associated with the ricin intoxication, which will lead to a better understanding of the pathogenic mechanism in ricin and a new way to develop the preventive and treatment measures on ricin intoxication.

## 3. Experimental Section

### 3.1. Animals

Six-week-old BALB/c female mice were purchased from the Center of Experimental Animals, Jilin University, Changchun, China. Mice were housed with a 12-h light-dark cycle and fed with standard diet ad libitum. All research protocols were approved by the Institutional Animal Care and Use Committee at Institute of Military Veterinary.

### 3.2. Establishment of Animal Models of Poisoning

0.9% NaCl solution (saline) was prepared, autoclaved at 121 °C for 20 min, and then used as the aerosol solvent. 0.9% NaCl solution without ricin was used on the control group, and ricin-containing saline (ricin concentration at 0.1 mg/mL) was used on the experimental group. Aerosol Generator (TSI3079, TSI incorporated, St. Paul, MN, USA) was connected to Aerosol Diluter (3302A, TSI incorporated, St. Paul, MN, USA) and Aerodynamic Particle Sizer (APS3321, TSI incorporated, St. Paul, MN, USA). The flow rate was 200 L/h, and the aerosol generation time was 240 s. 30 s after the initiation of aerosol generation, the Particle Sizer was switched on and samples were collected. Samples were collected for 20 s and diluted 1:20 with the Aerosol Diluter. The process was repeated five times. Experiments were performed at 25 °C with humidity of 60%. The Ricin exposure time for experiment subjects was 30 min.

### 3.3. Histology Analysis of Lung Tissues

Animals were sacrificed at 12, 24 and 48 h unless otherwise indicated. After dissection, lung tissues were stored in 4% paraformaldehyde solution for 24 h, then dehydrated and embedded in paraffin. For histologic analysis, 5 μm tissue slices were mounted on glass slides. The resulting slides were deparaffinized and stained with H&E according to the following standard procedure [31]. Then the slides were observed and photographed under Optical Microscope (Olympus BH, Tokyo, Japan).

### 3.4. Protein Sample Preparation and 2-DE

Lung tissue samples from the Control group and Experimental group (48 h post-exposure) were crushed using liquid nitrogen and suspended in lysis buffer (9.5 M urea, 4% CHAPS, 65 mM DTT, and 0.2% carrier ampholyte) which contained a protease inhibitor cocktail (Roche, Mannheim, Germany). The suspension was homogenized, sonicated on ice and centrifuged at 14,000 rpm for 1 h at 4 °C. The supernatant was collected and the protein concentration was determined using a Protein Assay kit (Bio-Rad, Richmond, VA, USA). Protein aliquots equivalent to 100 mg were stored at −80 °C for future use.

2-DE (Bio-Rad) was used to separate proteins as described by De-la-Pena C *et al.* [32]. The 2-DE gels were scanned using a GS-710 imaging densitometer and the digitized images were analyzed with the PDQuest software (Bio-Rad).

### 3.5. Mass Spectrometry and Peptide Mass Fingerprint

Protein spots were cut from the gels, then destained and washed. The spots were kept in 0.2 M NH_4_HCO_3_ for 20 min and then lyophilized. Each spot was digested overnight with 12.5 ng/mL trypsin in 0.1 M NH_4_HCO_3_, and the digested proteins were extracted three times with 50% ACN, 0.1% TFA solution. All mass spectra date were acquired on an AutoFlex MALDI-TOF/TOF mass spectrometer with LIFT technology (Bruker Daltonics, Bremen, Germany). MS/MS data were acquired with a N_2_ laser at a 25-Hz sampling rate [33]. PMF and MS data were combined using FlexAnalysis and the combined data set was submitted to MASCOT [34,35] for protein identification. The MASCOT result is a Probability-based Mowse Score [36] which is expressed as −10 × log(*p*), where *p* is the probability. Protein scores greater than 63 are considered statistically significant (*p* < 0.05). The above process was repeated three times. The criteria for selecting protein spots were (i) spots showing greater than twofold expression changes between control group and experimental group; (ii) reproducible spots which were detected in all three replicate experiments; and (iii) successful identification of spots using MALDI-TOF analysis.

## 4. Conclusions

The present study developed an aerosolized ricin-poisoned mouse model. With proteomic analysis, we found that six proteins, including Apoa1 apolipoprotein, Ywhaz 14-3-3 protein, Prdx6 Uncharacterized Protein, Selenium-binding protein 1, HMGB1, and DPYL-2 occurred at different levels in lung tissue pre-and post-exposure to aerosolized ricin. These proteins might be the key proteins related to ricin poisoning. Further studies will focus on clarifying the mechanism and the roles for these proteins in the ricin-poisoning process.

## Figures and Tables

**Table 1.** The numerical values of protein spots with differential protein expressions were analyzed using PDQuest software. Standard spot (SSP) numbers are numbers assigned to each protein spot by PDQuest software, and each SSP number uniquely identifies one protein. Experimental group, Group E; Control group, Group C.

SSP	Group C	Group E
2102	968.3	23,189.7
3002	1396.1	5797.8
3101	2488.1	22,238.7
3402	12,325.5	5535.4
4002	5161.5	536.2
4303	8417.6	11,741.3
4401	11,423.8	5873.1
5101	—	350.5
5502	1433.7	—
5601	31,398.6	7768.7
6103	50,965.5	29,575.2
6502	31,905.5	11,282.5
6701	31,537.9	4494.9
7304	9879.2	3170.5

**Table 2.** MALDI-TOF/MS identification of differentially expressed proteins. Selected protein spots were obtained using EXQuest Spot Cutter (BIO-RAD), with the proteins enzymatically hydrolyzed and identified using MALDI-TOF/MS. Proteins with score of 100, *i.e.*, 100% homology were included. ↑ higher expression, ↓ lower expression.

SSP	ID	NAME	*M*_W_ (KDa)	PI	Difference
2102/3101	IPI00877236	Apoa1 apolipoprotein A-1 preproprotein	30.6	5.51	↑
3002	IPI00116498	Ywhaz 14-3-3 protein zeta/delta	28	4.73	↑
3402/4401/6502	IPI00131695	Alb Serum albumin	70.7	5.75	↓
4002	IPI00420261	Hmgb1 High mobility	24.9	5.62	↓
5502	IPI00623845	Selenium-binding protein 1	53.05	5.87	↓
6103/5101	IPI00758024	Prdx6 Uncharacterized Protein	25	5.98	↓
6701	IPI00114375	Dihydropyrimidinase related protein 2	62.64	5.95	↓
7304	IPI00127596	Creatine kinase M-type	43.25	6.58	↓
